# A pediatric case of tick‐bite–Induced meat allergy and recall urticaria

**DOI:** 10.1002/ccr3.4773

**Published:** 2021-09-24

**Authors:** May Saleem, Caroline Nilsson

**Affiliations:** ^1^ Sachs’ Children and Youth Hospital Stockholm Sweden; ^2^ Clinical Science and Education Södersjukhuset Karolinska Institute Sachs’ Children and Youth Hospital Stockholm Sweden

**Keywords:** alpha‐gal syndrome, delayed anaphylaxis to red meat, re‐call urticaria, red meat allergy, tick bite

## Abstract

Alpha‐gal syndrome should be suspected also in children in case of typical allergic symptoms in the evening or night during tick seasons and the event of recall urticaria. It is, however, still a challenge for both patients and clinicians.

## Graphical abstract

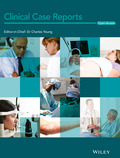

## 1 BACKGROUND

Here, we report a pediatric case of a boy who has been sensitized to alpha‐gal after tick bites. He had delayed hypersensitivity reactions including anaphylaxis and recall urticaria phenomenon in the sites of the previous tick bites. We want to increase the awareness that the alpha‐gal syndrome exists also in children.

Meat from any kind of mammal can cause an allergic reaction and relates to at least three different forms of mammalian meat allergy (MMA): primary beef allergy, pork‐cat syndrome, and alpha‐gal syndrome.^1^ MMA has historically been considered uncommon.^2^ However, more cases have been reported in the recent years and the numbers continue to rise—partially due to increased recognition of the diagnosis.^3^ MMA diagnosis relies on a combination of clinical history and measurement of specific IgE in serum.^2^ The management includes counseling patients on dietary avoidance, education, and emergency plans.^1^, ^3^

*Primary beef allergy* is predominantly an IgE‐mediated disease with immediate allergic reactions (within 2 h after intake of the allergen) mainly in young atopic children but occurs in rare cases also in adults.^1^, ^2^ The best‐characterized allergen in primary beef allergy is bovine serum albumin Bos d 6 (a major allergen in beef), but also Bos d 7 (bovine immunoglobulin).^1^ The protein Bos d 6 is found in muscle tissue and milk, which explains why many patients with a primary allergy to beef also have reactions when consuming milk.^3^ In a study of 28 children with primary beef allergy, 93% were also allergic to milk. On the contrary, when milk‐allergic children have been examined, only 20% have reported a meat allergy. Many of these children grow out of their meat allergy in primary school.^3^ The allergic symptoms can be mild to severe. Individuals may experience itching in the mouth and throat, urticaria, gastrointestinal symptoms, and anaphylaxis.^1^, ^2^, ^3^

*Pork‐cat syndrome* may affect cat allergic individuals who develop IgE‐ab against cat albumin, Fel d 2. When sensitized to Fel d 2, allergic symptoms may occur in connection with the consumption of pork.^3^ This is because IgE‐ab against Fel d 2 can cross‐react with porcine albumin, Sus s 1.^3^ Sometimes, there is also a cross‐reaction with albumin found in beef, Bos d 6, and then, allergic symptoms can also occur after consuming beef. Well‐cooked pork seems more likely to be tolerated and relates to the fact that pork serum albumin is heat labile.^3^ Those affected are usually adolescents or young adults.^3^ Most individuals with pork‐cat syndrome are or have been cat owners and the route of sensitization goes via the respiratory tract.^3^ The allergic symptoms usually start within an hour after eating pork and can be mild to severe.^1^, ^3^

*Alpha‐gal syndrome* is characterized by a delayed symptom onset (within 2–24 h).^2^, ^3^, ^4^ Allergic reactions to red meat typically occur in the night, several hours after a meat dinner and can lead to various allergic symptoms.^4^ The allergy is caused by IgE‐ab to the galactose‐alpha‐1, 3‐galactose (alpha‐gal), a carbohydrate found in glycolipids and glycoproteins in mammals but lacking in humans and higher primates.^5^ Alpha‐gal is present in muscles, blood cells, parenchymal tissue, and secretion of non‐primate mammals and allergic reaction can occur upon exposure to wide range of mammalian products (e.g., meat, kidneys, milk, gelatin, and blood products).^3^, ^6^ The route of IgE sensitization is through human skin via tick bites (species, *Ixodes ricinus*, in Europe). Alpha‐gal molecules will be transmitted into the human skin when bitten by a tick and an immunological response starts and some individual will develop IgE‐antibodies to alpha‐gal.^2^, ^7^, ^8^ Many individuals with alpha‐gal syndrome know that they have had one or more tick bites some months before the first allergic reaction.^8^ This form of allergy usually develops in adulthood but also occurs in children.^4^, ^9^

The first reaction usually appears in the summer or autumn, and the symptoms commonly reported include urticaria, abdominal cramping, gastrointestinal symptoms, and anaphylaxis.^7^, ^10^ Because patients typically do not get symptoms every time, they eat mammalian meat or intestinal food, it can be difficult to diagnose the alpha‐gal syndrom.^10^ The reactions can also occur after consuming dairy products and gelatin but are usually milder than those triggered by mammalian meat.^10^ Alpha‐gal is heat stable, and reaction can also occur after consuming well‐cooked meat.^1^, ^7^

Cases of delayed anaphylactic reactions after consumption of red meat among adults was first recognized in the United State 2009.^5^, ^11^, ^12^ Previously, there have been a few cases reported among children, but reports have now come from United States, Turkey, and Italy.^5^, ^13^

“Recall urticaria,” a memory response at the site of the previous tick bite, was recently reported among adults with alpha‐gal syndrome.^14^ This case report presents a Swedish 12‐year‐old boy with alpha‐gal syndrome and highlights the recall urticaria phenomena.

## 2 CASE DESCRIPTION

A 12‐year‐old boy was referred to the allergy department at Sachs´ Children and Youth Hospital in Stockholm with a medical history of mild asthma and cat allergy but no previous food allergy. The boy has previously always eaten a normal diet. He visited the emergency department (ED) due to recurrent allergic reactions and anaphylaxis about 3–4 times a year since 2014, always in the autumn season (August –December). His parents reported that he had several tick bites months before the onset of symptoms. The most common symptoms at arrival to ED were anaphylaxis (asthma attack, general urticaria, itching in the palms and soles of the feet, swollen tongue, eyes, and lips) and distinct swelling and redness behind the ears and in the inguinal region. Allergic reactions were mostly in the evening or in the night. The boy also had recurrent discomfort with constipation, stomach pain, and vomiting.

The family lives in the countryside and the boy has been bitten by ticks many times which were mostly situated behind his ears and in the inguinal region. Various investigations due to his symptoms from the gastrointestinal tract, including gastroscopy, have been carried out without explanation of his symptoms. Systemic mastocyosis, cat‐pork syndrome, celiac disease, lactose intolerance, and eosinophilic esophagitis have been excluded. Both lactose‐ and gluten‐free diets have been tested without improvement, and he was treated with laxative regularly. He was referred to a pediatric allergist for management; however, there were no clinical findings at the physical examination inclusive spirometry without signs of impaired lung function. The blood analyses showed an increased level of total IgE 910–1090 kU/L (ref <124 kU/L)) and increased levels of specific IgE‐ab to cat and alpha‐gal, Table 1. He also had specific‐IgE‐ab to other food and airborne allergens, however, at a low level, Table 1. Serum tryptase were measured at some of the anaphylaxis events and showed 8.2, 2.9, and 4.5 µg/L, respectively (ref <11.4 µg/L).

**TABLE 1:** Specific IgE with the detection level 0.1 kU/L

Allergens	kU/L
Alpha‐Gal	25.0
Beef	6.2
Cow´s Milk	1.1
Pork	3.1
Birch	0.2
Cat	47.0
Dog	3.4
Dust Mite	0.5
Horse	0.3
Cat nFel d2	<0.1
Chicken	<0.1
Egg	<0.1
Nuts	<0.1
Peanuts	<0.1
Soy	<0.1
Mushroom	<0.1
Fish	<0.1
Wheat	<0.1
Shrimp	<0.1
Grass	<0.1
Mold	<0.1

MMA was suspected after the results of specific IgE‐ab to alpha‐gal which was clearly elevated (25 kU/L, ref <0.1 kU/L), and the boy was recommended a diet without meat from mammals. He got a clear improvement in his symptoms after the withdrawal of red meat. However, stomach pain and vomiting as well as urticaria behind his ears and in the inguinal region, which was the site of his previous tick bites, still occurred when he consumed cow milk or sweets with gelatin from mammal animals. After the boy was given a diet with strict avoidance of meat from mammals, cow´s milk, and gelatin, he had no further symptoms.

## 3 DISCUSSION

To our knowledge, this is the first published case in the pediatric population with allergy to mammal meat induced by tick bites and recall urticaria phenomenon. With this case report, we want to increase the awareness that the alpha‐gal syndrome also exists in children living in areas where ticks are common, although this syndrome is known to mostly affect adults.

Identification of alpha‐gal cases may not be straight forward and a challenge for both patients and clinicians, especially when it presents with delayed anaphylaxis, angioedema, and urticaria that occurs several hours after eating a meal.^10^, ^11^, ^15^ Another aggravating circumstance can be that allergic symptoms may not be present every time when eating mammalian meat.^10^ Also, IgE‐ab to alpha‐gal can be present without any symptoms at all.^3^, ^14^, ^16^, ^17^ It is therefore of most importance to take a careful medical history.

Recall urticaria is a rare biological phenomenon. It has been known as a clinical sign since its first description by Kelso et al. in 1994^18^ and characterized by urticaria only at the skin area where an allergen has previously been injected and when the patient is re‐exposed with the same allergen but from another source, for example, through the gastrointestinal tract.^19^ In this case, it appeared when the boy had eaten red meat, cow milk, or gelatin containing alpha‐gal. He then got urticaria at the previous tick bite sites besides his other allergic symptoms. This urticaria phenomenon is, however, quite common in patients undergoing subcutaneous allergen‐specific immunotherapy (SCIT),^18^, ^20^ characterized by hives only at previously injected site when the patients are re‐exposed.^19^ Recall urticaria phenomenon in the medical history strengthens the diagnosis in patients with clinical suspicion of alpha‐gal syndrome.^2^, ^14^

In conclusion, alpha‐gal syndrome should be suspected also in children in case of typical allergic symptoms in the evening or night, especially children who lives in tick‐rich areas or previously got tick bites, and in the event of recall urticaria. For those who reacts to meat, milk, and gelatin, it is important to also be careful with drugs and vaccines containing gelatin as a stabilizer.^21^

## CONFLICT OF INTEREST

May Saleem declare no conflict of interest. Caroline Nilsson reports grants to institution and advisory board fees from Aimmune Therapeutics, a Nestlé Health Science company, and Novartis, as well as lecture fees from MEDA, ALK, Thermo Fisher, and GSK.

## AUTHOR CONTRIBUTIONS

May Saleem and Caroline Nilsson participated in design of the case report and manuscript writing and read and approved the final manuscript.

## ETHICAL APPROVAL

This case report is not published or submitted elsewhere before nor plagiarized, falsified, or fabricated. Approval has been obtained from guardian via personal communication. The collected information and data do not include patient identity.

## Data Availability

The data supporting this case report's findings are available from the corresponding author upon reasonable request.
